# Drought stress introduces growth, physiological traits and ecological stoichiometry changes in two contrasting *Cunninghamia lanceolata* cultivars planted in continuous-plantation soils

**DOI:** 10.1186/s12870-021-03159-3

**Published:** 2021-08-18

**Authors:** Fangyuan Bian, Yukui Wang, Baoli Duan, Zhizhuang Wu, Yuanbing Zhang, Yufang Bi, Anke Wang, Hao Zhong, Xuhua Du

**Affiliations:** 1 Key Laboratory of National Forestry and Grassland Administration on Bamboo Resources and Utilization, China National Bamboo Research Center, Zhejiang 310012 Hangzhou, China; 2 Chengdu Institute of Biology, Chinese Academy of Sciences, 610041 Chengdu, Sichuan China; 3 Key Laboratory of Mountain Surface Processes and Ecological Regulation, Institute of Mountain Hazards and Environment, Chinese Academy of Sciences, 610041 Chengdu, China

**Keywords:** *Cunninghamia lanceolata*, drought stress, physiological traits, ecological stoichiometry, continuous-plantation soil, super cultivar

## Abstract

**Background:**

The decrease in *Cunninghamia lanceolata* (Lamb.) production on continuously planted soil is an essential problem. In this study, two-year-old seedlings of two cultivars (a normal cultivar, NC, and a super cultivar, SC) were grown in two types of soil (not planted (NP) soil; continuously planted (CP) soil) with three watering regimes, and the interactive effects on plant growth and physiological traits were investigated in a greenhouse experiment. The water contents of the soil in the control (CK) (normal water content), medium water content (MWC) and low water content (LWC) treatments reached 75−80 %, 45−50 % and 20−25 % of the field water capacity, respectively.

**Results:**

The results indicated that the CP soil had a negative effect on growth and physiological traits and that the LWC treatment caused even more severe and comprehensive negative effects. In both cultivars, the CP soil significantly decreased the height increment (HI), basal diameter increment (DI), dry matter accumulation (DMA), net photosynthetic rate (Pn), total chlorophyll content (TChl), carotenoid content (Caro) and photosynthetic nitrogen use efficiency (PNUE). Compared to the NP soil, the CP soil also decreased the proline and soluble protein contents, nitrogen use efficiency (NUE) and phosphorus use efficiency (PUE) and increased the nitrogen:phosphorus ratio in roots, stems and leaves. The LWC treatment decreased growth and photosynthesis, changed ecological stoichiometry, induced oxidative stress, promoted water use efficiency and damaged chloroplast ultrastructure. Significant increases in ascorbate peroxidase (APX), peroxidase (POD), soluble protein and proline contents were found in the LWC treatment. Compared with the NC, the SC was more tolerant to the CP soil and water stress, as indicated by the higher levels of DMA, Pn, and WUE. After exposure to the CP soil and watering regimes, the decreases in biomass accumulation and gas exchange were more pronounced.

**Conclusions:**

The combination of drought and CP soil may have detrimental effects on *C. lanceolata* growth, and low water content enhances the impacts of CP soil stress on *C. lanceolata* seedlings. The superiority of the SC over the NC is significant in Chinese fir plantation soil. Therefore, continuously planted soil can be utilized to cultivate improved varieties of *C. lanceolata* and maintain water capacity. This can improve their growth and physiological performance to a certain extent.

**Supplementary Information:**

The online version contains supplementary material available at 10.1186/s12870-021-03159-3.

## Background

Plantation forests have increased global forest cover. However, there is ample evidence of the negative effects of plantation forests on ecosystem functions [1–3]. One of the major problems is that plantation forests generally have degraded soil qualities after long-term successive planting, with lowered N availability to plants [4]. Indeed, plants grown in continuous-plantation soils have reduced growth vigor and plant productivity, which can alter the structure of soil microbial communities and affect microbial groups [5]. Because of this, the dominant tree height has declined by 7−23 % in continuous-plantation soils of Chinese fir plantations [6]. In addition, drought is becoming a more serious problem in many regions [7, 8], and severe droughts can have potentially unexpected impacts on crop production in parts of China [9]. Drought-stressed plants showed lower gas exchange rates, upregulation of antioxidant enzymatic systems and accumulation of osmolytes [10–12]. The utilization of fertilizers is affected by the water content in the soil [13]. In nature, plants are generally exposed to a combination of drought and degraded soil. The responses of plants to the interaction of environmental stresses is not the same as the responses of plants to each individual stressor [14]. Therefore, studies of the impact of combinations of stressors are of considerable significance.

The decline in productivity with continuous cropping in Chinese fir ecosystems has caused much concern and is a crucial problem that needs to be solved. Chinese fir (*Cunninghamia lanceolata* (Lamb.) Hook) is an endemic, evergreen coniferous species that is cultivated as a commercial tree. Due to its advantageous features, such as fast growth, pest and disease resistance, and high timber quality, it is one of the most important timber tree species in the tropics and subtropics of China. Chinese fir plantations have been widely established in South China [15]. Continuous cropping of Chinese fir has caused soil degradation, resulting in reduced productivity [16–18]. Improved varieties have been widely and predominantly used in agriculture [19, 20]. Studies have shown that rice varieties improved for application in coastal environments have better tolerance to the abiotic stresses prevailing in coastal agroecosystems and provide higher and stable yields [19]. Research on improved varieties of Chinese fir has shown that the stand basal area growth of third-generation seeds of young Chinese fir plantations is significantly higher than that of first-generation seeds [21].

In this study, two-year-old seedlings of *C. lanceolata* cultivars (a normal cultivar, NC, and a super cultivar, SC) were grown in two soil types (soil from land not planted with Chinese fir (NP soil) and soil from land continuously planted with Chinese fir, CP soil) with three watering regimes. We investigated the cultivar-specific responses of *C. lanceolata* in terms of growth, gas exchange, photosynthetic pigments, osmotic adjustment, reactive oxygen species (ROS), enzymatic antioxidants and ultrastructural integrity in the CP soil, drought, and their combinations. Here, we addressed the hypothesis that growth and physiological traits would be decreased by consecutive monocultures in Chinese fir plantations. We also tested the hypothesis that the super cultivar would be less affected than the normal cultivar by drought and continuously planted soil. Moreover, we proposed that an interaction between drought and continuously planted soil would be present. More specifically, we aimed (1) to determine whether and which growth and physiological traits of *C. lanceolata* are affected by exposure to continuously planted soil, drought and their combination and (2) to evaluate whether the water condition can enhance the effects of continuously planted soil.

## Results

### Water, cultivar and CP soil effects on growth, photosynthesis and chlorophyll content

Overall, *C. lanceolata* in the unplanted soil (NP) showed higher increments of height (HI), basal diameter (DI) and dry mass accumulation (DMA) than plants grown in the soil with continuously planted (CP) *C. lanceolata* (Table 1). In the two cultivars, HI, DI and DMA showed a significant decrease in response to water stress and CP soil alone, and a much more dramatic decrease occurred when LWC and CP were applied together. The R/S ratio was affected by the interaction of cultivar and CP soil; *C. lanceolata* had a lower R/S ratio under the LWC treatment than under the MWC and CK treatments. In both cultivars, the LWC treatment significantly increased the total chlorophyll content and carotenoid content in the two soil types. The chlorophyll a/b ratio was unaffected by either cultivar or CP soil, while water stress significantly increased the chlorophyll a/b ratio.

**Table 1:** Height increment (HI), basal diameter increment (DI), dry mass accumulation (DMA), root to shoot ratio (R/S), total chlorophyll content (TChl), chlorophyll a/b ratio (Chl_a/b_), carotenoid content (Caro) in two cultivars with two-year-old seedlings of *C.lanceolata*as affected by water, soil and their interaction

Cultivars	Water condition	Soil	HI (cm)	DI (mm)	DMA (g)	R/S	TChl	Chl_a/b_	Caro
NC	CK	NP	26.03 ± 1.64a	5.42 ± 0.75ab	33.20 ± 4.13b	0.43 ± 0.06ab	0.96 ± 0.08d	2.45 ± 0.03bc	0.23 ± 0.00d
NC	CK	CP	16.13 ± 1.87de	3.34 ± 0.38e	14.29 ± 0.83e	0.41 ± 0.06b	0.92 ± 0.03d	2.47 ± 0.05b	0.22 ± 0.02d
NC	MWC	NP	19.75 ± 1.54 cd	4.26 ± 0.61 cd	19.23 ± 1.97d	0.44 ± 0.05ab	1.53 ± 0.00c	2.81 ± 0.06a	0.31 ± 0.06bc
NC	MWC	CP	11.79 ± 1.32f	3.10 ± 0.42e	9.15 ± 1.32 g	0.49 ± 0.01a	1.60 ± 0.19c	2.85 ± 0.13a	0.28 ± 0.02bc
NC	LWC	NP	14.49 ± 1.31ef	3.72 ± 0.36d	11.92 ± 1.69f	0.30 ± 0.02 cd	2.68 ± 0.34a	2.79 ± 0.17a	0.40 ± 0.02a
NC	LWC	CP	8.08 ± 1.03 g	3.11 ± 0.6e	7.63 ± 0.8 h	0.28 ± 0.03d	2.36 ± 0.17b	2.76 ± 0.02a	0.32 ± 0.05bc
SC	CK	NP	25.38 ± 1.35ab	5.59 ± 0.87a	41.42 ± 4.29a	0.40 ± 0.05b	1.06 ± 0.01d	2.22 ± 0.16c	0.25 ± 0.01 cd
SC	CK	CP	21.98 ± 1.93bc	4.78 ± 0.44bc	25.25 ± 2.65c	0.46 ± 0.05ab	0.85 ± 0.00d	2.40 ± 0.05bc	0.24 ± 0.02d
SC	MWC	NP	20.22 ± 1.63 cd	5.05 ± 0.22ab	28.43 ± 1.28bc	0.34 ± 0.04c	1.50 ± 0.04c	2.75 ± 0.13a	0.29 ± 0.02bc
SC	MWC	CP	17.92 ± 1.49de	4.77 ± 0.58bc	17.63 ± 1.51d	0.44 ± 0.05ab	1.68 ± 0.15c	2.72 ± 0.11a	0.30 ± 0.03bc
SC	LWC	NP	15.38 ± 1.28ef	4.73 ± 0.48bc	18.73 ± 1.29d	0.27 ± 0.02d	2.72 ± 0.09a	2.57 ± 0.16ab	0.33 ± 0.04ab
SC	LWC	CP	7.60 ± 1.02 g	3.59 ± 0.27de	8.37 ± 0.99gh	0.34 ± 0.03c	1.71 ± 0.02c	2.75 ± 0.04a	0.35 ± 0.11ab
		P:Fw	< 0.0001	< 0.0001	< 0.0001	< 0.0001	< 0.001	0.0004	< 0.0001
		P:Fc	0.0013	< 0.0001	< 0.0001	0.2782	0.0671	0.5350	0.9392
		P:Fs	< 0.0001	< 0.0001	< 0.0001	0.0380	0.0197	0.1746	0.2561
		P:Fw×Fc	0.7955	0.2412	0.0373	0.0623	0.0086	0.8434	0.4706
		P:Fw×Fs	0.6754	0.0885	< 0.0001	0.3968	0.0013	0.3346	0.7508
		P:Fc×Fs	0.0031	0.0569	0.4922	0.0365	< 0.0001	0.0769	0.0810
		P:Fw×Fc×Fs	0.4997	0.0269	0.1861	0.9037	< 0.0001	0.2423	0.73892

Values are mean ± SE. Values followed by different small letters and within parameters are significantly different at the *P* < 0.05 level according to Tukey’s test. NC, normal cultivar; SC, super cultivar; CK, control watering regime; MWC, mild water stress; LWC, heavy water stress; Fw, water stress effect; Fc, cultivar effect; Fs, soil effect; Fw×Fc, water and cultivar interaction; Fs×Fc, soil and cultivar interaction; Fw×Fs, water and soil interaction; Fw×Fc×Fs, soil, water and cultivar interaction

In the two cultivars, the *Pn*, *gs*, E and PNUE were all significantly decreased when the seedlings were exposed to the MWC or LWC treatments (Table 2). Compared with the NP soil, the seedlings of both cultivars in the CP soil had lower *Pn*, E and PNUE, and *Ci* values. All these parameters were significantly affected by the interaction between water and soil type.

**Table 2:** Net photosynthesis rate (*Pn*), stomatal conductance (*gs*), transpiration (*E*), photosynthetic N use efficiency (PNUE), intercellular carbon dioxide concentration (*Ci*) in two cultivars with two-year-old seedlings of *C. lanceolata* as affected by water, soil and their interaction

Cultivars	Water condition	Soil	*Pn*(µmol m^− 2^ s^− 1^)	*gs*(mol m^− 2^ s^− 1^)	*Ci* (µmol mol^− 1^)	*E* (mmol m^− 2^ s^− 1^)	PNUE (µmol g^− 1^ s^− 1^)
NC	CK	NP	4.38 ± 0.44a	0.09 ± 0.03b	267.06 ± 20.67c	0.88 ± 0.11ab	4.52 ± 0.45a
NC	CK	CP	3.02 ± 0.45b	0.12 ± 0.01a	320.06 ± 7.39a	0.73 ± 0.09c	4.28 ± 0.62a
NC	MWC	NP	2.97 ± 0.29b	0.06 ± 0.01c	298.63 ± 5.61b	0.77 ± 0.10bc	2.97 ± 0.28 cd
NC	MWC	CP	1.80 ± 0.24f	0.05 ± 0.01d	330.50 ± 14.37a	0.58 ± 0.09d	2.18 ± 0.29e
NC	LWC	NP	2.25 ± 0.12de	0.03 ± 0.01e	165.32 ± 2.81f	0.34 ± 0.03e	2.70 ± 0.14 cd
NC	LWC	CP	1.69 ± 0.22f	0.02 ± 0.00f	265.73 ± 21.75c	0.17 ± 0.05f	2.24 ± 0.30e
SC	CK	NP	4.53 ± 0.74a	0.10 ± 0.02b	285.85 ± 27.63b	0.93 ± 0.05a	4.17 ± 0.68ab
SC	CK	CP	2.99 ± 0.40b	0.10 ± 0.03ab	327.32 ± 7.07a	0.85 ± 0.18a	3.09 ± 0.41c
SC	MWC	NP	2.50 ± 0.18 cd	0.06 ± 0.01c	294.12 ± 1.62b	0.54 ± 0.04d	2.63 ± 0.19d
SC	MWC	CP	2.80 ± 0.28bc	0.06 ± 0.01 cd	293.37 ± 2.94b	0.55 ± 0.04d	3.82 ± 0.38b
SC	LWC	NP	2.29 ± 0.35d	0.03 ± 0.00e	214.71 ± 14.39e	0.23 ± 0.05f	2.77 ± 0.41 cd
SC	LWC	CP	1.94 ± 0.10ef	0.03 ± 0.00ef	251.94 ± 22.11d	0.22 ± 0.06f	2.62 ± 0.14d
		P:Fw	< 0.0001	< 0.0001	< 0.0001	< 0.0001	< 0.0001
		P:Fc	0.0188	0.6076	0.2204	0.4886	0.46
		P:Fs	< 0.001	0.4783	0.0019	< 0.0019	< 0.0001
		P:Fw×Fc	0.2662	0.2611	< 0.0001	< 0.0001	< 0.0001
		P:Fw×Fs	< 0.001	< 0.0001	< 0.0001	0.0010	< 0.0001
		P:Fc×Fs	0.00098	0.94	< 0.0001	0.05	0.0022
		P:Fw×Fc×Fs	< 0.001	0.0798	< 0.0001	< 0.0001	< 0.0001

Values are mean ± SE. Values followed by different small letters and within parameter are significantly different at the *P* < 0.05 level according to Tukey’s test

In the two cultivars, the LWC significantly increased the WUEi, Wp, δ^13^C, CUE, NUE and PUE in *C. lanceolata* planted in the two soil types (Table 3). In addition, for the normal cultivar, seedlings exposed to mild water stress had lower WUEi, δ^13^C, CUE, NUE and PUE than those of plants in the CK, but this was not the case for the super cultivar. The experiment had a three-factor random design, namely, 2 cultivars × 3 water conditions × 2 types of soil. When considering any two of these factors, significant effects were detected in WUEi, Wp, δ^13^C, NUE and PUE.

**Table 3:** Instant use efficiency (WUEi), water potential (Wp), foliar carbon isotope composition (δC^13^), carbon use efficiency (CUE), nitrogen use efficiency (NUE), phosphorus use efficiency (PUE) in two cultivars with two-year-old seedlings of *C. lanceolata* as affected by water, soil and their interaction

Cultivars	Water condition	Soil	WUEi	Wp (MPa)	δC^13^	CUE	NUE	PUE
NC	CK	NP	4.98 ± 0.70d	-0.86 ± 0.05d	-28.33 ± 0.15c	2.45 ± 0.28de	3.06 ± 0.07de	2.37 ± 0.16e
NC	CK	CP	4.13 ± 0.94e	-0.84 ± 0.10d	-28.52 ± 0.23c	2.68 ± 0.31 cd	3.04 ± 0.06de	2.71 ± 0.36d
NC	MWC	NP	3.86 ± 0.15ef	-0.89 ± 0.10d	-28.97 ± 0.52d	2.23 ± 0.12de	2.98 ± 0.02e	2.66 ± 0.31de
NC	MWC	CP	3.24 ± 0.86f	-1.26 ± 0.05bc	-28.70 ± 0.29 cd	2.18 ± 0.04e	2.40 ± 0.02 g	2.39 ± 0.31de
NC	LWC	NP	6.67 ± 0.73c	-1.17 ± 0.16c	-27.25 ± 0.13a	3.00 ± 0.22bc	3.47 ± 0.14bc	3.07 ± 0.36 cd
NC	LWC	CP	10.61 ± 1.04a	-1.63 ± 0.18a	-27.78 ± 0.18b	3.90 ± 0.28a	3.51 ± 0.09b	4.60 ± 0.77a
SC	CK	NP	4.91 ± 0.50d	-0.96 ± 0.09d	-28.57 ± 0.08 cd	2.55 ± 0.20cde	3.12 ± 0.03d	3.15 ± 0.44c
SC	CK	CP	3.58 ± 0.42f	-0.97 ± 0.13d	-28.70 ± 0.30 cd	2.22 ± 0.06de	2.76 ± 0.00ef	2.36 ± 0.30de
SC	MWC	NP	4.65 ± 0.18de	-0.96 ± 0.05d	-28.55 ± 0.39 cd	2.98 ± 0.19bc	3.23 ± 0.24 cd	3.51 ± 0.46bc
SC	MWC	CP	5.09 ± 0.12d	-1.33 ± 0.08bc	-28.26 ± 0.22c	2.33 ± 0.21de	2.54 ± 0.11 fg	2.04 ± 0.13e
SC	LWC	NP	10.15 ± 0.62ab	-1.40 ± 0.07b	-27.37 ± 0.17a	3.19 ± 0.06b	3.66 ± 0.22b	3.78 ± 0.29b
SC	LWC	CP	9.60 ± 1.28b	-1.75 ± 0.16a	-27.70 ± 0.31b	3.65 ± 0.10a	4.00 ± 0.02a	3.37 ± 0.38bc
		P:Fw	< 0.0001	< 0.0001	< 0.0001	< 0.0001	< 0.0001	0.0002
		P:Fc	0.03	0.6465	0.07	0.6201	0.097	0.003
		P:Fs	0.46	0.0030	0.0006	0.00576	< 0.0001	0.0369
		P:Fw×Fc	0.0008	0.5172	0.0061	0.01	< 0.0001	0.2845
		P:Fw×Fs	< 0.0001	0.7467	0.0.4	0.0184	< 0.0001	0.0167
		P:Fc×Fs	0.04	< 0.0001	0.59	0.0004	0.01	0.0007
		P:Fw×Fc×Fs	< 0.0001	0.0001	0.0267	0.2484	0.0005	0.3965

Values are mean ± SE. Values followed by different small letters and within parameters are significantly different at the *P* < 0.05 level according to Tukey’s test

### Water, cultivar and CP soil effects on the C:N ratio and N:P ratios in leaves, stems and roots

In the normal cultivar, the C:N ratios of the roots and stems of plants in the CP soil were significantly decreased under water stress (Table 4). In addition, soil type affected the C:N ratios of the roots, stems and leaves. This indicated that the C:N ratios of the stems and leaves were higher in the CP soil than in the NP soil. Compared with those in the leaves, the C:N ratios in the stems and roots were higher in all the treatments. Regarding the N:P ratio, significant increases induced by the CP soil were detected in all the organs in both cultivars (Table 4). In both cultivars, there were significant increments in the N:P ratio of the roots and leaves when exposed to the MWC and LWC treatments. Soil type had a significant effect on the N:P ratios of all organs.

**Table 4:** Effects of water stress on stoichiometry changes in *C. lanceolata* planted on different continuous plantation soils

Cultivars	Water condition	Soil	Root		Stem		Leaf	
			C:N	N:P	C:N	N:P	C:N	N:P
NC	CK	NP	61.47 ± 4.53ab	4.18 ± 0.08f	75.21 ± 1.15bc	4.45 ± 0.21e	36.75 ± 0.81 cd	5.11 ± 1.14e
NC	CK	CP	59.38 ± 6.36ab	8.82 ± 0.11cde	92.00 ± 8.75ab	8.19 ± 0.19c	38.38 ± 2.88c	9.22 ± 1.39de
NC	MWC	NP	53.72 ± 0.78ab	5.67 ± 0.04f	62.95 ± 0.31c	4.40 ± 1.30e	31.86 ± 0.92d	9.47 ± 0.82de
NC	MWC	CP	57.29 ± 5.52ab	17.60 ± 1.20a	81.09 ± 0.94b	12.44 ± 1.60a	40.82 ± 4.08bc	18.43 ± 0.80a
NC	LWC	NP	49.25 ± 3.03b	7.03 ± 0.28ef	62.88 ± 1.53c	5.51 ± 0.17de	35.30 ± 2.85 cd	9.51 ± 0.58de
NC	LWC	CP	51.40 ± 0.42b	15.14 ± 0.02ab	82.93 ± 3.64ab	10.92 ± 0.14ab	49.39 ± 1.82a	10.60 ± 5.08bc
SC	CK	NP	57.07 ± 6.50ab	5.51 ± 0.16f	77.03 ± 0.32bc	4.86 ± 0.19de	34.90 ± 1.22 cd	5.99 ± 1.99e
SC	CK	CP	66.49 ± 4.76a	10.93 ± 1.18de	87.52 ± 4.77ab	9.47 ± 0.68bc	39.06 ± 0.77bc	15.02 ± 0.33b
SC	MWC	NP	58.71 ± 4.61ab	5.84 ± 0.72f	61.98 ± 2.13c	6.22 ± 0.20d	35.26 ± 1.63 cd	9.83 ± 0.14cde
SC	MWC	CP	66.27 ± 7.35a	11.98 ± 1.17bcd	99.19 ± 7.85a	10.14 ± 0.90b	44.81 ± 0.57ab	18.49 ± 1.87a
SC	LWC	NP	49.86 ± 4.75b	7.59 ± 0.26de	80.69 ± 17.77b	5.80 ± 0.09de	45.07 ± 5.77ab	6.89 ± 1.32e
SC	LWC	CP	66.99 ± 3.15a	12.77 ± 0.51bc	83.51 ± 12.48ab	9.73 ± 0.48bc	50.25 ± 2.95a	12.92 ± 4.67 cd
		P:Fw	0.0765	0.0155	0.1447	0.0020	0.0001	< 0.0001
		P:Fc	0.0295	0.4654	0.0718	0.8546	0.0239	0.4864
		P:Fs	0.0150	< 0.0001	< 0.0001	< 0.0001	< 0.0001	< 0.0001
		P:Fw×Fc	0.4377	0.1390	0.4152	0.1867	0.1135	0.0029
		P:Fw×Fs	0.5494	0.1927	0.1209	0.0632	0.0455	0.0041
		P:Fc×Fs	0.0409	0.1443	0.9489	0.0180	0.3911	0.1370
		P:Fw×Fc×Fs	0.5990	0.3173	0.0863	0.0138	0.1117	0.1373

Values are mean ± SE. Values followed by different small letters and within parameter are significantly different at the *P* < 0.05 level according to Tukey’s test

### Water, cultivar and CP soil effects on enzyme activity, soluble protein content and proline content

**Fig. 1:**
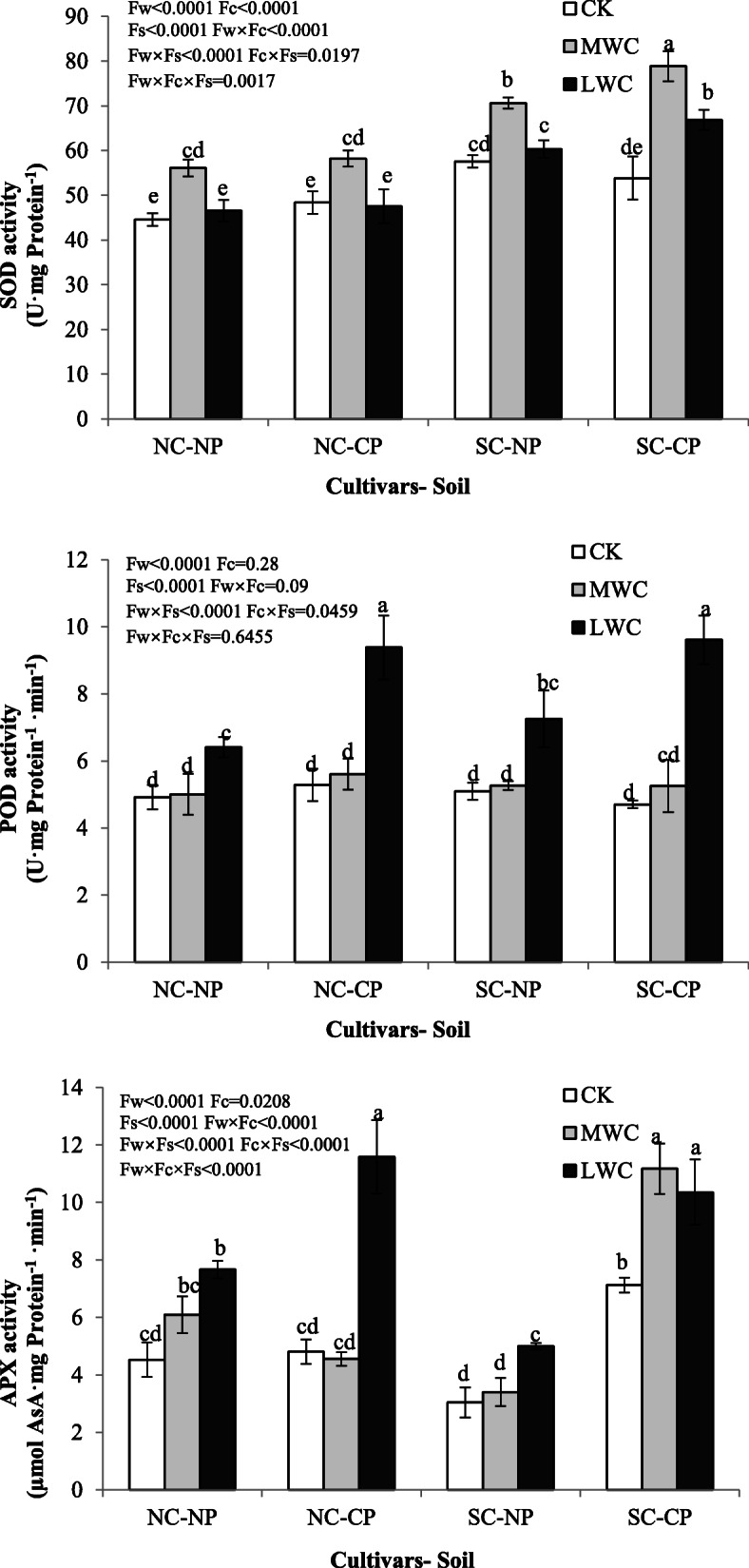
Antioxidant enzyme activities of *C. lanceolata* under different treatments. NC, normal cultivar; SC, super cultivar; CK, control watering regime; MWC, mild water stress; LWC, heavy water stress. Values are mean ± SE Values followed by different capital letters are significantly different at the *P* < 0.05 level according to Tukey’s test

Compared with that in the CK, the APX and POD activity significantly increased in the LWC treatment. In contrast, the SOD increased only in the super cultivar in the CP soil (Fig. 1). In addition, SOD activity was higher in *C. lanceolata* plants in the MWC treatment than in plants in the CK and LWC treatments.

**Fig. 2:**
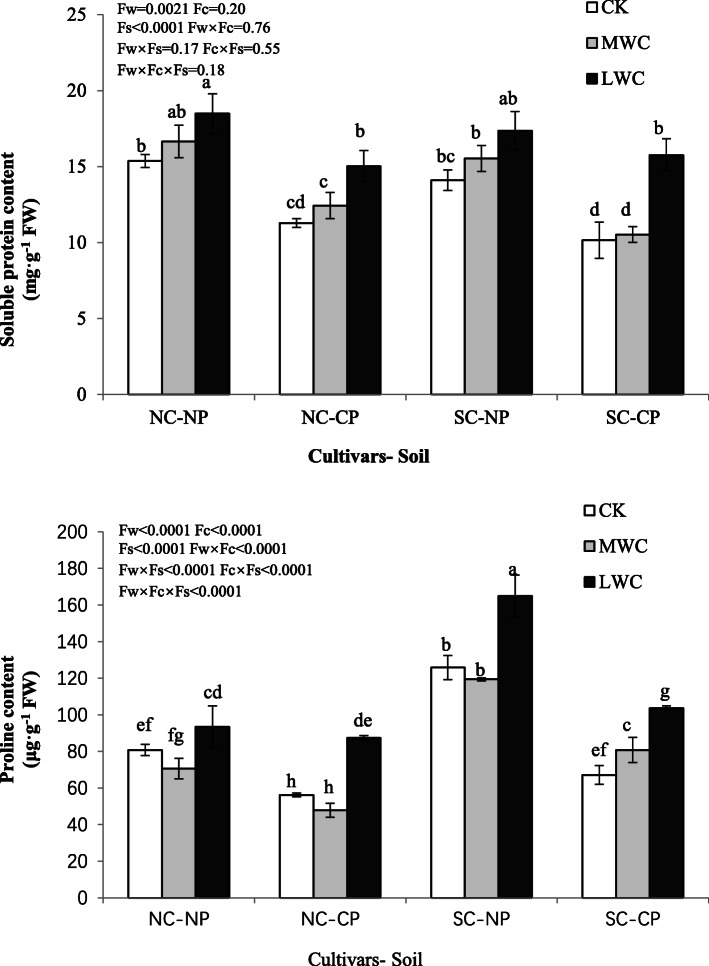
Soluble proteins and proline contents of *C. lanceolata* under different treatments. NC, normal cultivar; SC, super cultivar; CK, control watering regime; MWC, mild water stress; LWC, heavy water stress. Values are mean ± SE. Values followed by different capital letters are significantly different at the *P* < 0.05 level according to Tukey’s test

The seedlings planted in the NP soil showed higher soluble protein contents than plants in the CP soil, and the same pattern was observed for the proline content (Fig. 2). In both cultivars, plants in the LWC treatment had increased soluble protein and proline contents. In addition, there was little difference between the two cultivars.

### Water, cultivar and CP soil effects on mesophyll cells

**Fig. 3:**
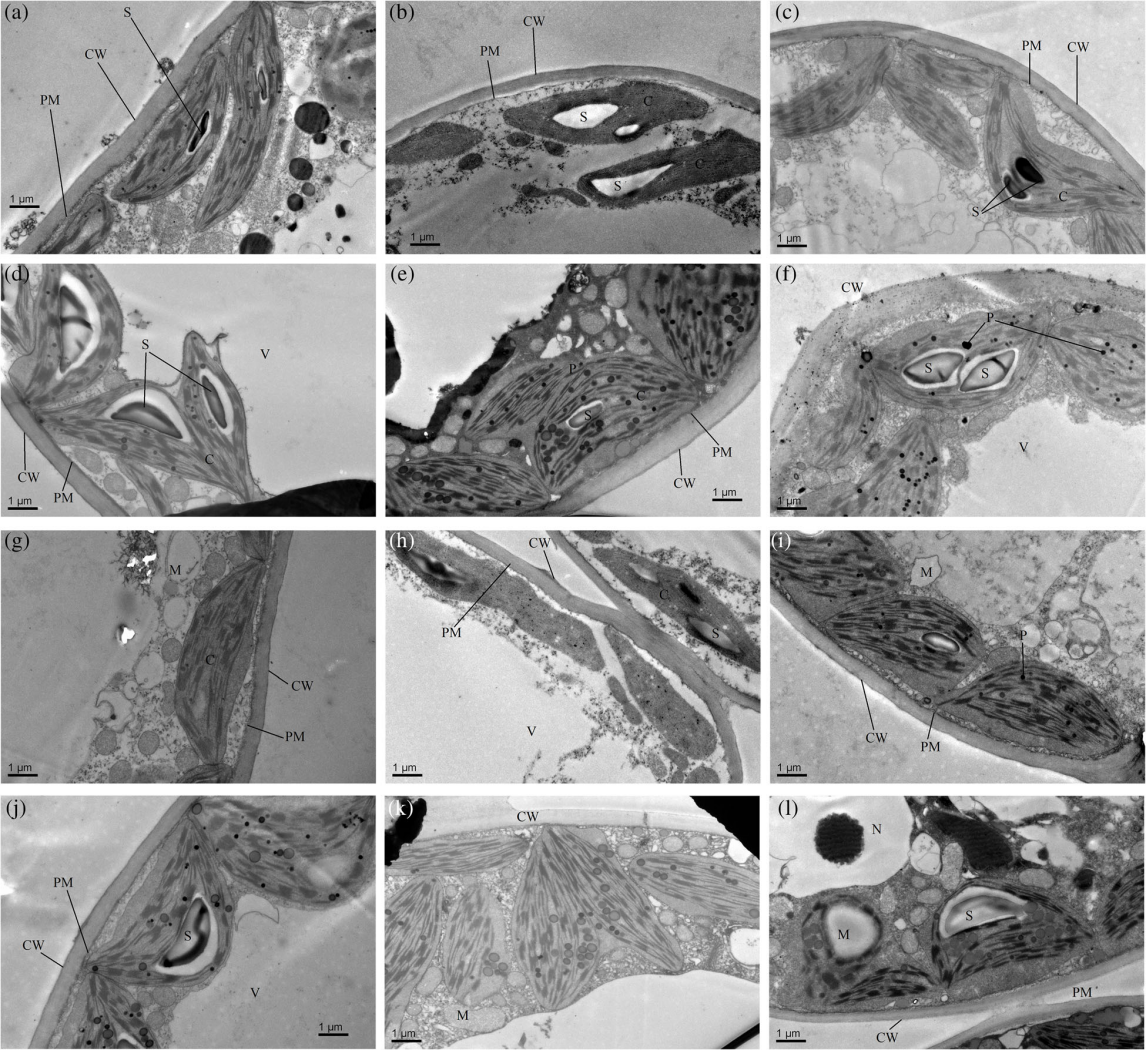
Transmission electron microscopy observations of mesophyll cells in *C. lanceolata* planted in different continuous plantation soils. NC, normal cultivar; SC, super cultivar; CK, control watering regime; MWC, mild water stress; LWC, heavy water stress. Values are mean ± SE. Values followed by different capital letters are significantly different at the *P* < 0.05 level according to Tukey’s test. NC-CK-NP (A), NC-CK-CP (B), NC-MWC-NP (C), NC-MWC-CP (D), NC-LWC-NP (E), NC-LWC-CP (F), SC-CK-NP (A), SC-CK-CP (B), SC-MWC-NP (C), SC-MWC-CP (D), SC-LWC-NP (E), SC-LWC-CP (F). The bar corresponds to 1 μm. C, chloroplast; PM, plasma membrane; CW, cell wall; P, plastoglobulus; S, starch granule; N, nucleus; NC, nucleolus; V, vacuole

*C. lanceolata* in the CK and MWC treatments had smooth, clean and continuous cell membranes and cell walls (Fig. 3A−D, G−J). However, alterations in the ultrastructure of Chinese fir leaves under different treatments were detected by TEM analysis. Compared with leaves of plants in the NP soil, leaves of plants in the CP soil had more starch granules. The LWC treatment alone seriously affected the chloroplasts of both cultivars in the two types of soil, especially the NC-CP-LWC (Fig. 3F) and SC-NP-LWC (Fig. 3K) treatments.

## Discussion

### Physiological responses of growth, photosynthesis and chlorophyll content in response to drought among seedlings planted in continuous-plantation soil

This study examined the hypotheses that growth and physiological responses would be significantly degenerated by consecutive monocultures in *C. lanceolata* plantation soils. In our study, CP soil caused a decrease in height increment, basal diameter increment, dry matter accumulation (DMA), net photosynthetic rate (Pn) and photosynthetic nitrogen use efficiency (PNUE). Similarly, previous studies have reported that productivity declines were observed in Chinese fir plantations under long-term monoculture [17, 22]. The CP soil suppressed growth and leaf photosynthesis, as shown in other studies [23]; there are three possible reasons for this finding: The depletion of nutrient elements, soil acidification and a reduction in microbial biomass [16, 24, 25]. Generally, soil degradation in the continuous cropping system of our study appeared to be less severe than expected based on previous reports. The soil properties of the samples from the Chinese fir forest in this study showed that the pH of the CP soil was lower than that of the NP soil. In the CP soil, total P and available P showed a tendency to decrease compared with those in the NP soil. Soil acidification could be a problem for *C. lanceolata* grown in continuous cropping ecosystems [26]. Soil acidification and available P deficiency appear to be two of the important factors leading to a decline in *C. lanceolata* production under continuous rotation practices.

Pant biomass decreased significantly in the two cultivars affected by drought stress, similar to the results of previous studies reported for other plant species [27, 28]. This effect might be due to decreases in photosynthesis and chlorophyll content [29]. Similarly, the decrease in the contents of C in the leaves under drought conditions showed that plant growth was affected by a low net photosynthetic rate. Drought affected the reduction in biomass productivity and significantly reduced plant physiological traits such as gas exchange parameters, consistent with many previous studies [30, 31]. In this study, drought stress increased WUE_i_ and δ^13^C. The production and accumulation of soluble protein in leaves may be caused by the effects of stress on nitrogen reserves. Compared with the normal cultivar, the super cultivar exhibited higher biomass production and greater water use efficiency when exposed to drought, indicating that the super cultivar is more tolerant to drought. Long-term nitrogen use efficiency (NUE) can be estimated using the C:N ratio [32]. The observed decrease in the C:N ratio under drought and in the CP soil indicates that the combinations of these two stressors reduced the NUE, which was mainly caused by the decrease in C content and the increase in N content.

### Cellular damage and enzymatic activities

Drought may enhance the scavenging capacity of reactive oxygen species [33]. Drought can trigger membrane damage. SOD, APX and POD are components of the plant antioxidative defense system. Our results indicated that seedlings exposed to drought conditions showed high levels of APX and POD antioxidant enzymes. Similarly, the activities of several antioxidant enzymes, such as APX and SOD, were found to increase under the effects of drought [34, 35]. Proline accumulation is generally observed in plants exposed to drought stress [36]. Soluble protein and free proline contents were significantly increased by drought in both cultivars (Fig. 2); this could contribute to osmotic adjustment. Affected by the interaction between water and cultivar, the long-term water use efficiency evaluated by δ^13^C changed obviously, and the δ^13^C of the super cultivar in the LWC treatment was significantly higher than that of the normal cultivar in the MWC and CK treatments. Compared to the normal cultivar, the super cultivar showed higher dry matter accumulation and accumulated much more free proline for osmotic adjustment. The super cultivar also had a more efficient antioxidant system, with higher activities of SOD than the normal cultivar. According to the effects of drought on the growth parameters, free proline contents and WUEi, the super cultivar was concluded to have higher drought tolerance than the NC.

Previous results indicated that the mesophyll cell ultrastructure of both cultivars showed damage to the nuclear envelope and the membranes of mitochondria, thylakoids and stromata under drought treatment, consistent with our results. In addition, this effect was more obvious in the CP soils than in the NP soils. These breakdowns in cell membranes may be due to an increase in ROS accumulation in different cell compartments, since ROS have an important role in inner membrane system organization.

### Growth and physiological responses to the combination of drought and CP soil

Under field conditions, plants are often affected by many different abiotic stress factors simultaneously. Plant responses to different combinations of stressors are unique [37]. This study investigated the effects of the interaction of drought and CP soil on growth and physiological traits. Plant height increment and biomass production were more severely affected by the combination of stressors than by drought alone. When the CP soil was combined with drought, the drought-induced growth reduction was most obvious. Moreover, the intensive damage to the ultrastructure caused by drought × CP confirmed that the effects of antioxidation in the CP soil were modified by the water supply. When plants were grown in the CP soil, there was little difference in plant growth, POD activity and free proline contents under water sufficiency and drought stress, which indicated that the CP soil may enhance certain effects under water stress conditions.

## Conclusions

The performance (tree height increment, diameter increment and biomass) of the super cultivar was better than that of the normal cultivar when soil water was abundant, and the LWC and CP soil treatment caused damage to plant growth, photosynthesis and other physiological indicators as well as to the ultrastructure of mesophyll cells. These results suggest that low water content aggravates the impact of soil stress from CP soil on *C. lanceolata* seedlings, and the superiority of the super cultivar over the normal cultivar is significant in the Chinese fir plantation soil.

## Methods

### Plant materials and experimental design

Two-year-old seedlings were obtained from Yangkou Forest Farm, Fujian Province. First-generation seedlings were obtained for the normal cultivar, and third-generation seedlings were obtained for the super cultivar. Seedlings with relatively consistent height and diameter were planted in 30-L plastic pots. The seedlings were grown in a controlled environment room at Zhejiang A & F University (N30°23′, E119°72′), China. Before the trial started, to maintain seedling growth, we irrigated the pots once every three days or when there was a need. Approximately four months later, the trial was performed from March to October. All materials were obtained with permission.

The experimental design consisted of a completely randomized design, with 12 factorial combinations of the two Chinese fir cultivars (NC and SC), two types of soil (NP soil and CP soil), and three levels of water stress. This trial consisted of three replications per treatment and five plants per replication (plastic pot). Each pot was planted with one Chinese fir seedling. The SC has stronger growth in height and diameter than the NC [38]. The sapling stage of the SC, i.e., the period before trees are available for timber production, is shorter than that of NC. The performance of the SC in of Chinese fir plantations on clear-cut forestlands was significantly better than that of sprouting trees [39]. However, the ability of the SC to adapt to drought stress and its performance in logging areas has been less studied. The NP soil was from forestlands not planted with Chinese fir, and most of the trees in this forestland were subtropical species of evergreen broad-leaved trees. The CP soil was from a Chinese fir forest that had been replanted more than 20 years after the first fir harvest. The properties of the NP soil and CP soil used in this study are shown in Table 5 (based on kg^-1^ dry soil).

**Table 5:** The properties of the non-continuous planting soil and continuous planting soil from the forest land of *C. lanceolata*

Soil type	NP	CP
pH	5.47	4.63
Total N (g kg^− 1^)	1.21	0.92
Hydrolysable N (mg kg^− 1^)	162.07	127.08
Total P (g kg^− 1^)	0.61	0.43
Available P (mg kg^− 1^)	2.1	1.53
Total K (g kg^− 1^)	13.69	10.41
Organic matter (g kg^− 1^)	36.36	28.22

Soil moisture content was controlled to remain within a certain range by the weight method. According to the known soil weight (approximately 30 kg), moisture content and maximum field capacity, the weight of the target treatment in the control area was calculated, and water management was carried out regularly. The maximum field water capacity of the test soil was 28−29 %. There were three drought treatments: a control (watered and maintained at 75−80 % field water capacity, normal water control, CK), medium drought stress (watered and maintained at 45−50 % field water capacity, medium water content, MWC), and heavy drought stress (watered and maintained at 20−25 % field water capacity, low water content, LWC). Water was added when the percentage water content was outside the specified level at 16:00−18:00 every 3 d by the weight method.

During the experiment, the potted seedlings of each test treatment were periodically rotated to minimize the influence of growth factors within each treatment. During the last 10 d of this trial, the protective enzyme activity, content of lipid peroxidation materials, relative electrolyte conductivity, gas exchange, chlorophyll fluorescence and chlorophyll pigment content were all evaluated. After harvesting, samples were separated into leaves, stems and roots. The samples were used for measurements of the water content, biomass, total carbon (C), total nitrogen (N) and total phosphorus (P) in different organs.

### Assays of height growth, biomass and water content

Initially, we measured the height and biomass of 30 plants per cultivar. The samples were separated into different organs for biomass assays. This process was repeated at the end of the trial. The increases in biomass, height growth and water content were calculated by subtracting the final data from the mean initial data. Water content was calculated as the fresh weight minus the dry weight. The root-to-shoot ratio was calculated with the root biomass and shoot biomass. Biomass was obtained by oven-drying at 60 °C until a constant weight was reached [40].

### Assays of nutrient biological stoichiometry and nutrient use efficiency

The contents of C, N and P in roots and leaves were analyzed and determined. The N and P contents in different organs were measured by the micro-Kjeldhal and molybdenum antimony colorimetric methods, respectively [40, 41]. The C content was measured by an elemental analyzer (vario MAX cube CNS, Elementar, Germany). Nutrient biological stoichiometry in leaves, stems and roots was calculated as the N content/P content. C use efficiency (CUE), N use efficiency (NUE) and P use efficiency (PUE) were calculated by the ratios of nutrient contents in organs above the soil and organs below the soil [42]. These ratios were used to evaluate the allocation of nutrients in plants.

### Determination of enzyme activity and lipid peroxidation

Fresh leaf samples from the third or fourth expanded leaves were collected for enzyme extraction. The samples were collected and put into boxes with ice bags. Enzymes were extracted at 4 °C from approximately 0.2 g leaf samples with 100 mM phosphate buffer (pH 7.8). This buffer contained 0.1 mM MEDTA, 1 % (v/v) polyvinyl pyrrolidone (PVP), 0.1 mM phenyl methyl sulfonyl fluoride (PMSF) and 0.2 % (v/v) Triton X-100. The extraction solutions were centrifuged at 6,000 r/min for approximately 30 min. The supernatants were used for the measurements of superoxide dismutase (SOD; EC 1.15.1.1), peroxidase (POD; EC 1.11.1.11) and soluble proteins.

SOD activity was assayed by the inhibition of the photochemical reduction of β-nitro blue tretrazolium chloride (NBT) [41, 43], and SOD was measured at 560 nm with a spectrophotometer (Shimadzu UV-2550, Kyoto, Japan). POD activity was measured with guaiacol as the substrate at 470 nm [44]. Malondialdehyde (MDA) content is an essential parameter to illustrate the lipid peroxidation level, and this was determined via the thiobarbituric acid method (TBA) [41]. The total soluble protein content was determined by the Coomassie brilliant blue staining method [41]. The APX enzyme activity was determined with ascorbic acid.

The proline content was determined by the acid ninhydrin occlusion method [45]. To a 0.20 g leaf sample, 5 ml 3 % sulfosalicylic acid solution was added; the solution was covered, extracted in a boiling water bath for 15 min (shaking frequently during the extraction), and then filtered in a clean test tube after cooling. The filtrate is the extract of proline. After obtaining the extract, the measurement was carried out according to the literature.

### Gas exchange

We selected five samples from the third or fourth fully expanded and exposed young leaves for gas exchange and chlorophyll fluorescence measurements.

Gas exchange was measured with the LI-COR 6400XT portable photosynthesis system (LI-CORBiosciences, Inc., Lincoln, USA) on sunny days from 8:30 to 11:00. In this system, the ambient CO_2_, light intensity, relative humidity and temperature were controlled at 400 µmol·mol^-1^, 1200 µmol·m^-2^·s^-1^, 60 % and 28 °C, respectively. CO_2_ was provided by a steel CO_2_ cylinder specifically for the LI-COR 6400XT. The light was provided by an LED red-blue light chamber. The leaves in the chamber were collected, and the area was measured with a leaf area analysis system (CI202, CID, USA).

The photosynthetic N use efficiency (PNUE) was calculated as the ratio between the photosynthetic rate and leaf N concentration per area [33].

### Water use efficiency

The instantaneous water use efficiency (WUE_i_) was determined by the ratio of maximum carbon assimilation at saturating light (A_max_) to the leaf transpiration rate (E). Five leaf samples per replication were selected for the measurement of water potential (Wp) with a pressure tank (3005, Soilmositure Equipment Corp, USA). These measurements were done before sunrise, from 2:00–4:30.

Five leaf samples per treatment were selected for the measurement of δ^13^C using an isotope ratio mass spectrometer (Delta V Advantage, Thermo Fisher Scientific, Inc., USA) combined with an elemental analyzer (Flash EA1112 HT, Thermo Fisher Scientific, Inc.). The samples were burned at high temperature to generate CO_2_ in the elemental analyzer. We measured the ratio of ^13^ C content to ^12^ C content using a mass spectrometer and calculated the ratio of δ^13^C after comparison with the international standard substance (Pee Dee Belemnite, PDB).

### Chlorophyll content and ultrastructure of chloroplasts

Samples were collected for each treatment for chlorophyll content determination. The harvested Chinese fir leaves were quickly washed and dried, and the midrib was cut. Each sample was then cut into a 0.2 cm filament or a small piece and evenly mixed. The samples were weighed to the nearest 0.1 g and placed in a test tube, and 8 ml of 95 % ethanol was added. The leaves were soaked in the dark for 24 h and shaken three to four times in the middle of the period until they turned white [45].

Five samples per treatment were selected to examine the ultrastructure of chloroplasts using a transmission electron microscope (Hitachi H-7650, Hitachi, Ibaraki, Japan). The samples were processed as follows. (1) Double fixation: specimens were fixed with 2.5 % glutaraldehyde in phosphate buffer (0.1 M, pH 7.0) for more than 4 h, washed three times (for 15 min each time) in the phosphate buffer (0.1 M, pH 7.0), then post-fixed with 1 % OsO_4_ in the phosphate buffer (0.1 M, pH 7.0) for 1–2 h and washed three times (for 15 min each time) in the phosphate buffer (0.1 M, pH 7.0). (2) Dehydration: specimens were dehydrated by a graded series of ethanol (30 %, 50 %, 70 %, 80 %, 90 %, 95 and 100 %) for approximately 15−20 min at each step and then transferred to absolute acetone for 20 min. (3) Infiltration: specimens were placed in a 1:1 mixture of absolute acetone and the final Spurr resin mixture for 1 h at room temperature and then transferred to a 1:3 mixture of absolute acetone and the final resin mixture for 3 h and to the final Spurr resin mixture overnight. (4) Embedding and ultrathin sectioning: specimens were placed in Eppendorf tubes containing Spurr resin and heated at 70 °C for more than 9 h. The specimens were sectioned with a LEICA EM Uc7 ultratome, and sections were stained with uranyl acetate and alkaline lead citrate for 5−10 min and observed using a Hitachi model H-7650 TEM.

### Statistical analyses

Data were analyzed by the MANOVA procedure in the Statistical Analysis System (SAS). The cultivar types, soil treatments and water availability were analyzed as the main factors. Multiple comparisons were made using Duncan’s test at a significance level of α = 0.05.

## Supplementary information

Additional file 1: **Table S1**. Effects of water stress on nitrogen contents changes of *C. lanceolata* in different continuous plantation soils.

## Data Availability

The relevant procedures and information can not be made public for the time being but are available from the corresponding author on reasonable request.

## Abbreviations

NC: normal cultivar
SC: super cultivar
NP: not planted
CP: continuously planted
CK: normal water content
MS: medium stress
HS: heavy stress
CUE: C use efficiency
NUE: N use efficiency
PUE: P use efficiency
SOD: superoxide dismutase
NBT: nitro blue tretrazolium
TBA: thiobarbituric acid
MDA: malondialdehyde
POD: peroxidase
PNUE: photosynthetic N use efficiency
WUE_i_: instantaneous water use efficiency
Wp: water potential
DMA: dry matter accumulation
Pn: net photosynthetic rate
HI: Height increment
DI: basal diameter increment
R/S: root to shoot ratio
TChl: total chlorophyll content
Chl_a/b_: chlorophyll a/b ratio
Caro: carotenoid content
E: transpiration
Ci: intercellular carbon dioxide concentration
δC^13^: foliar carbon isotope composition
