# Supplementary material for: Drought stress introduces growth, physiological traits and ecological stoichiometry changes in two contrasting Cunninghamia lanceolata cultivars planted in continuous-plantation soils
Source: BMC Plant Biol. 2021 Aug 18;21:379. doi: 10.1186/s12870-021-03159-3 (PMC8371764; doi:10.1186/s12870-021-03159-3)
Supplement: Supplementary file 1 — Table S1. Effects of water stress on nitrogen contents changes of C. lanceolata in different continuous plantation soils. [file 12870_2021_3159_MOESM1_ESM.docx]

**Table S1** Effects of water stress on nitrogen contents changes of *C. lanceolata* in different continuous plantation soils

| Cultivars | Water condition | Soil | Root | Stem | Leaf |
| --- | --- | --- | --- | --- | --- |
| NC | CK | NP | 8.03 ± 0.01 | 7.07 ± 0.46 | 12.89 ± 0.17 |
| NC | CK | CP | 7.40 ± 0.01 | 5.92 ± 0.89 | 12.23 ± 0.54 |
| NC | MWC | NP | 8.95 ± 0.00 | 7.79 ± 0.68 | 14.43 ± 0.31 |
| NC | MWC | CP | 7.91 ± 0.11 | 6.29 ± 0.56 | 11.08 ± 0.01 |
| NC | LWC | NP | 10.50 ± 0.23 | 7.62 ± 0.43 | 13.01 ± 0.58 |
| NC | LWC | CP | 8.80 ± 0.01 | 6.02 ± 0.12 | 9.57 ± 0.25 |
| SC | CK | NP | 8.57 ± 0.06 | 6.92 ± 0.76 | 12.92 ± 0.36 |
| SC | CK | CP | 7.31 ± 0.03 | 5.93 ± 0.06 | 11.74 ± 0.13 |
| SC | MWC | NP | 8.00 ± 0.10 | 7.62 ± 0.00 | 13.38 ± 0.33 |
| SC | MWC | CP | 6.94 ± 0.00 | 4.98 ± 0.66 | 9.96 ± 0.07 |
| SC | LWC | NP | 9.95 ± 0.13 | 5.96 ± 0.89 | 10.70 ± 0.20 |
| SC | LWC | CP | 7.61 ± 0.00 | 6.00 ± 0.31 | 10.12 ± 0.95 |
